# Cystatin F attenuates neuroinflammation and demyelination following murine coronavirus infection of the central nervous system

**DOI:** 10.1186/s12974-024-03153-0

**Published:** 2024-06-15

**Authors:** Amber R. Syage, Collin Pachow, Kaitlin M. Murray, Caden Henningfield, Kellie Fernandez, Annie Du, Yuting Cheng, Gema Olivarria, Shimako Kawauchi, Grant R. MacGregor, Kim N. Green, Thomas E. Lane

**Affiliations:** 1 Department of Neurobiology & Behavior, School of Biological Sciences, University of California, Irvine, 92697 USA; 2 Department of Molecular Biology & Biochemistry, School of Biological Sciences, University of California, Irvine, 92697 USA; 3 Transgenic Mouse Facility, ULAR, Office of Research, University of California, Irvine, 92697 USA; 4 Department of Developmental & Cell Biology, University of California, Irvine, 92697 USA; 5 Center for Virus Research, University of California, Irvine, 92697 USA

**Keywords:** Cystatin F, Coronavirus, Microglia, Demyelination, Remyelination

## Abstract

**Background:**

Cystatin F is a secreted lysosomal cysteine protease inhibitor that has been implicated in affecting the severity of demyelination and enhancing remyelination in pre-clinical models of immune-mediated demyelination. How cystatin F impacts neurologic disease severity following viral infection of the central nervous system (CNS) has not been well characterized and was the focus of this study. We used cystatin F null-mutant mice (*Cst7-/-*) with a well-established model of murine coronavirus-induced neurologic disease to evaluate the contributions of cystatin F in host defense, demyelination and remyelination.

**Methods:**

Wildtype controls and *Cst7-/-* mice were intracranially (i.c.) infected with a sublethal dose of the neurotropic JHM strain of mouse hepatitis virus (JHMV), with disease progression and survival monitored daily. Viral plaque assays and qPCR were used to assess viral levels in CNS. Immune cell infiltration into the CNS and immune cell activation were determined by flow cytometry and 10X genomics chromium 3’ single cell RNA sequencing (scRNA-seq). Spinal cord demyelination was determined by luxol fast blue (LFB) and Hematoxylin/Eosin (H&E) staining and axonal damage assessed by immunohistochemical staining for SMI-32. Remyelination was evaluated by electron microscopy (EM) and calculation of *g*-ratios.

**Results:**

JHMV-infected *Cst7-/-* mice were able to control viral replication within the CNS, indicating that cystatin F is not essential for an effective Th1 anti-viral immune response. Infiltration of T cells into the spinal cords of JHMV-infected *Cst7-/-* mice was increased compared to infected controls, and this correlated with increased axonal damage and demyelination associated with impaired remyelination. Single-cell RNA-seq of CD45 + cells enriched from spinal cords of infected *Cst7-/-* and control mice revealed enhanced expression of transcripts encoding T cell chemoattractants, *Cxcl9* and *Cxcl10*, combined with elevated expression of interferon-g (*Ifng)* and perforin (*Prf1*) transcripts in CD8 + T cells from *Cst7-/-* mice compared to controls.

**Conclusions:**

Cystatin F is not required for immune-mediated control of JHMV replication within the CNS. However, JHMV-infected *Cst7-/-* mice exhibited more severe clinical disease associated with increased demyelination and impaired remyelination. The increase in disease severity was associated with elevated expression of T cell chemoattractant chemokines, concurrent with increased neuroinflammation. These findings support the idea that cystatin F influences expression of proinflammatory gene expression impacting neuroinflammation, T cell activation and/or glia cell responses ultimately impacting neuroinflammation and neurologic disease.

**Supplementary Information:**

The online version contains supplementary material available at 10.1186/s12974-024-03153-0.

## Introduction

The human demyelinating disease, Multiple Sclerosis (MS), is a devastating neurodegenerative disease that affects millions of people worldwide. Though current therapies have been very successful in slowing the progression of disease in patients with the Relapsing-Remitting MS, there are currently few treatment options for individuals with progressive forms of MS, highlighting a fundamental need for discovery of treatments that can target inflammation and promote repair from within the CNS. To recapitulate the hallmark immune-mediated myelin damage seen in MS, we performed intracranial (i.c.) inoculation of C57BL/6J mice with the neurotropic JHM strain of mouse hepatitis virus (JHMV), which results in viral replication in glial cells with minimal infection of neurons [1, 2], leading to an acute encephalomyelitis and subsequent demyelination. Innate immune responses, including expression of type I interferon and chemokines that attract myeloid cells to the CNS, are important in limiting viral replication as well as allowing virus-specific T cells access to the brain parenchyma [3]. Both CD4 + and CD8 + T cells are recruited into the CNS of JHMV-infected mice by T cell chemotactic chemokines CCL5, CXCL9 and CXCL10 and these cells control viral replication through secretion of interferon-g (IFN-g) and cytolytic activity, respectively [4–11]. Antibody-secreting cells (ASCs) are also capable of responding to CXCL9 and CXCL10 and aid in host defense by preventing viral recrudescence [12, 13]. Nonetheless, sterile immunity is not achieved, and the majority of animals that survive the acute stage of disease develop immune-mediated demyelination in which both virus-specific T cells and macrophages amplify the severity of white matter damage associated with hind-limb paralysis [1, 2, 14, 15].

Under non-disease conditions, microglia function to maintain tissue homeostasis [16] and are important for maintaining overall myelin health through regulation of myelin growth and preservation of structural integrity [17, 18]. Microglia serve various functions under neuroinflammatory conditions by influencing antigen presentation, which either amplifies or restricts disease progression via secretion of regulatory factors that, in turn, control the activation state of inflammatory immune cells [19, 20]. Moreover, microglia are considered the immune cell of the CNS and are important in aiding in host defense in response to viral infection [21, 22]. Depletion of microglia via administration of small molecule antagonists specific for colony stimulating factor 1 receptor (CSF1R) results in increased susceptibility to viral-induced demyelinating disease [23–26]. Following microglia depletion, mice infected with JHMV exhibit an increase in mortality, associated with impaired control of viral replication within the CNS, that correlates with reduced expression of MHC class II on antigen-presenting cells (APCs) and muted CD4 + T cell anti-viral immune responses [27]. Further, the severity of spinal cord demyelination was increased while remyelination was dramatically reduced [28]. These findings support a role for microglia in regulating immune responses within the CNS in response to microbial infection as well as restricting the severity of white matter damage. In support of this, several recent reports demonstrated that microglia influence expression of genes and pathways associated with immune responses and myelin repair, in response to JHMV infection of the CNS [28–30]. We have shown that microglia depletion results in a dramatic reduction in transcripts encoding cystatin F (*Cst7*) that correlated with both increased demyelination and impaired remyelination [30].

Cystatin F is papain-like lysosomal cysteine protease inhibitor expressed by immune cells, including macrophages, cytotoxic T-lymphocytes (CTLs), and by microglia within the CNS, under conditions where demyelination and remyelination are concurrently occurring [31, 32]. Cystatin F is synthesized as an inactive dimer, which is converted to a monomeric form to enable inhibition of target cathepsins. Cystatins are generally found to act on cathepsins intracellularly or extracellularly; however, cystatin F is somewhat unusual in that it is secreted in an inactive form, only becoming active following internalization by neighboring cells, where it regulates cathepsin activity within the endosomes and lysosomes of those neighboring cells. The broad expression of cystatin F in immune cells raises the possibility that it has an important function in the immune response. Furthermore, microglial expression of cystatin F is triggered by the phagocytosis of myelin debris suggesting that cystatin F plays a role in response to demyelination [32]. Expression patterns of cystatin F overlap in demyelinating regions undergoing remyelination in pre-clinical mouse models of demyelination and in plaques from patients with multiple sclerosis (MS), garnering new interest in cystatin F for its potential impact on neuroinflammation, immune-mediated demyelination, and remyelination [31–34].

In this study, we set out to determine if cystatin F is involved in this process of modulating white matter damage and repair in a viral model of demyelinating disease and employed a novel *Cst7* knockout (*Cst7*-/-) mouse to assess the response to JHMV infection. Findings from this study indicate that the absence of cystatin F did not affect T cell-mediated control of JHMV replication within the CNS, arguing that this pathway is not critical in anti-viral effector responses. However, infiltration of T cells into the CNS of JHMV-infected *Cst7-/-* mice was increased early during chronic disease which correlated with increased expression of transcripts encoding T cell chemoattractant chemokines CXCL9 and CXCL10 accompanied by more severe demyelination, impaired remyelination, and elevated axonal damage. These results indicate a potential role for cystatin F as a mechanism influencing the severity of neuroinflammation, affecting both white matter damage and repair, in response to viral-induced neurologic disease.

## Materials and methods

### Generation and genotyping of *Cst7* null-mutant mice

To generate *Cst7*-null mutant mice (B6-*Cst7*^*em1Aduci*^/J, Jackson Laboratory stock number #037727), Alt-R Crispr RNAs (TMF1131–5’-CCTGCATTTCCCCAGCCATG-3’, TMF-1132–5’-AAGCAGAATGGCCAGCCACA-3’) and tracrRNA plus CAS9 protein (HiFi Cas9 nuclease V3, Integrated DNA Technologies (IDT), Coralville, IA) as a ribonucleoprotein (RNP) were microinjected into C57BL/6J zygotes (Jackson Lab Stock # 000664) to generate double stranded DNA breaks within exon 1 of *Cst7*. G0 founder mice were screened by PCR to identify animals having a deletion allele of *Cst7* produced by removal of DNA in the vicinity of the CAS9 cut sites, followed by non-homologous end joining (NHEJ) repair. G0 animals containing a prospective deletion allele were backcrossed with C57BL/6J mice, and N1F1 heterozygous mice were sequenced to determine the mutant allele. The *Cst7*-null allele (*Cst7*-null) has a deletion of 168 bp of contiguous DNA from nucleotide position 150,412,438 to 150,412,605 inclusive (nucleotide position from mouse genome build GRCm39, Ensembl release 107). The deletion begins one nucleotide upstream of the translation initiation ATG codon in exon 1 and extends 100 nucleotides into intron 1, i.e., exon 1 ATTTCCCCAGCcatgtggctgg…………catctttctTAGGACACTC intron 1, where the uppercase sequence is retained, and the lowercase sequence is deleted. N1F1 heterozygous *Cst7*^*em1Aduci*^ mutant mice were backcrossed to produce N2F1 heterozygotes, which were crossed to produce experimental *Cst7*-/- mice and wildtype (WT) controls (*Cst7*^+/+^). Oligonucleotides for PCR-based genotyping were purchased from IDT (Coralville, IA). *Cst7*^*em1Aduci*^ genotyping was performed using a common primer set to amplify both the *Cst7* wildtype allele and the *Cst7*^*em1Aduci*^ null allele (For 5′- CCCAAGTCCTGAAGATGAAGCG − 3′ and Rev 5′- AGGGTCTTTCTCAGGGTTCCA − 3′). Amplification was performed in a 10 µl reaction volume containing 2.5 ul diluted DNA, 1x New England Biolabs (NEB) PCR buffer, 0.75mM MgCl_2_, 0.2mM dNTPs, 0.25 μm each primer and 0.0375u Taq polymerase (NEB) for 30 cycles of 94 °C for 30 s, 55 °C for 30 s and 72 °C for 30 s. The wild-type allele produced a 344 bp product while the *Cst7*-null allele produced a 176 bp product. All mice used in this study were on an inbred or co-isogenic C57BL/6J background.

### Viral infection of mice

Cystatin F knock-out (*Cst7-/-*) mice were generated by the University of California, Irvine transgenic mouse facility. Five to eight-week-old male and female *Cst7*-/- mice and control (*Cst7+/+*) mice were infected i.c. with 1200 plaque forming units (PFU) of JHMV in 30µL of sterile Hanks balanced salt solution (HBSS), and animals were euthanized at days 7, 14, and 21 p.i. Clinical disease in JHMV-infected mice was evaluated using a previously described scale. To determine viral titers within brains, experimental animals were sacrificed at defined times p.i., with brains isolated and homogenized, and plaque assays performed on the DBT astrocytoma cell line as described previously [35]. Spinal cords of JHMV-infected controls and *Cst7*-/- mice were homogenized, with RNA extraction, cDNA synthesis, and qPCR for comparison of JHMV mRNA levels performed as previously described [36] Primer sequences used were: GAPDH – forward sequence, AACTTTGGCATTGTGGAAGG; reverse sequence, GGATGCAGGGATGATGTTCT: JHMV Matrix glycoprotein– forward sequence, TCAACCCCGAAACAAACAACC; reverse sequence, GGCTGTTAGTGTATGG TAATCCTCA. qPCR was performed using the Bio-Rad iQ5 and iTaq™ Universal SYBR® Green Supermix (Bio-Rad, Hercules, CA). Reactions were 10µL, and the machine was set to run for 1 cycle (95℃ for 3 min), followed by 40 cycles (95℃ for 10 s, then 55℃ for 30 s). Ct values for each sample were normalized to an internal control (GAPDH), yielding the dCt values; lower dCT values indicate higher mRNA levels while higher dCT values are indicative of lower mRNA levels as more cycles of amplification are required to detect signal. All animal studies were reviewed and approved by the University of California, Irvine Intuitional Animal Care and Use Committee.

### Cell isolation and flow cytometry

Flow cytometry was performed to identify inflammatory cells entering the CNS using established protocols [37]. In brief, single cell suspensions were generated from tissue samples by grinding with frosted microscope slides. Immune cells were enriched via a 2-step Percoll cushion (90% and 63%), and cells were collected at the interface of the two Percoll layers. Before staining with fluorescent antibodies, isolated cells were incubated with anti-CD16/32 Fc block (BD Biosciences, San Jose, CA) at a 1:100 dilution. Immuno-phenotyping of cells was performed using commercially available antibodies specific for the following cell surface markers: CD4 (Invitrogen, 11-0042-82), CD8a (Invitrogen, 17-0081-82), CD11b (Abcam, ab24874) and CD45 (BioLegend, 103,114; 103,130). Cells were simultaneously incubated with LIVE/DEAD Aqua Dead Cell Stain (Invitrogen, L34966). The following flow cytometric gating strategies were employed for inflammatory cells isolated from the CNS: macrophages/myeloid cells (CD45^hi^CD11b+) and microglia (CD45^lo^CD11b+); FITC-conjugated rat anti-mouse CD4 and a PE-conjugated tetramer specific for the CD4 immunodominant epitope present within the JHMV matrix (M) glycoprotein, spanning amino acids 133–147 (M133-147 tetramer), to determine total and virus-specific CD4 + cells [37, 38]; APC-conjugated rat anti-mouse CD8a and a PE-conjugated tetramer specific for the CD8 immunodominant epitope present in the spike (S) glycoprotein, spanning amino acids 510–518 (S510-518), to identify total and virus-specific CD8 + cells [37, 38]. Data were collected using a Novocyte flow cytometer and analyzed with FlowJo software (Tree Star Inc.).

### Histology

Mice were euthanized at days 14 and 21 p.i. with isoflurane, and immediately cardiac perfused with 20mL of 1X PBS, and spinal columns were isolated and stored in 4% paraformaldehyde at 4 °C for 24–36 h. The length of spinal cord extending from thoracic vertebrae 6–10 was carefully removed and cryoprotected in 30% sucrose at 4 °C for a minimum of 3 days, then cut into 1-mm transverse blocks and processed to preserve the craniocaudal orientation, and subsequently embedded in optimum cutting temperature (OCT) compound (VWR, Radnor, PA, USA). Spinal cord tissue was coronally cryosectioned at eight microns (µm) thick. Sections were stained with hematoxylin and eosin (H&E) in combination with luxol fast blue (LFB), with between 5 and 8 sections/mouse analyzed. Areas of total white matter and demyelinated white matter were determined with Image J Software and demyelination was scored as a percentage of total white matter from spinal cord sections analyzed [38–40].

### Immunofluorescence staining

Slides were washed extensively in 1X TBS, 0.2% Tween-20 and 0.2% Triton-X followed by antigen retrieval, an incubation with 1mM sodium citrate at 85^o^C for 3 min. Slides were stained with FluoroMyelin Green (Invitrogen, Cat#F3465, 1:300) for 20 min and washed again with 1X TBS. After blocking in 5% BSA (w/v) and normal goat serum for 1 h, samples were incubated in primary antibody, Anti-SMI-32 (nonphosphorylated neurofilament H; Biolegend, Cat# 801,701, 1:1000) overnight at 4 °C. Slides were washed in 1X TBS and incubated in secondary antibody, Alexa 594 goat anti-mouse (Invitrogen, Cat# A11005, 1:500) for 1 h at room temperature. Slides were mounted with Fluoromount-G with DAPI (Invitrogen, Cat#00-4959-52). Confocal images were taken with a 40X oil objective and 63X oil objective (SP8; Leica). Mean fluorescence intensity of axonal damage was measured using FIJI [41] and was quantified based on the reported integrated density of axonal damage normalized to the area of the selected region.

### Electron microscopy and analysis

Mice were euthanized at day 21 p.i. with isoflurane and immediately underwent cardiac perfusion with 20mL of 1X PBS followed by 20mL of 0.1 M cacodylate buffer containing 2% paraformaldehyde/2% glutaraldehyde. The length of spinal cord extending from thoracic vertebrae 6–10 was carefully removed and stored in 2% paraformaldehyde/2% glutaraldehyde overnight at 4 °C. Serial ultrathin sections of spinal cord embedded in Epon epoxy resin were stained with uranyl acetate-lead citrate and analyzed as previously described [37]. Images were taken at 1200X magnification in demyelinating regions within the spinal cord ventral and lateral funiculus for *Cst7-/-* mice (*n* = 6) and controls (*n* = 6). For each group, between 133 and 660 axons/mouse (7–20 images/mouse) were analyzed using Image J software. G-ratio was calculated as a ratio of axon diameter to total outer fiber diameter [36, 37]. Axons were considered remyelinated if myelin thickness was less than 0.5 μm and the *g*-ratio was greater than 0.65, and absence of myelin sheath was used as the criterion for demyelinated axons [42].

### 10X Genomics chromium single cell 3’ single cell RNA sequencing (scRNASeq)

Spinal cords were removed from uninfected *Cst7*-/- (n = 5) and control mice (n = 5), and JHMV-infected *Cst7*-/- (n = 6) and control mice (n = 6), at day 21 p.i. Cells were isolated from spinal cords via homogenization and Percoll gradient as previously described, keeping cells in RPMI with 10% FBS and on ice whenever possible. Isolated cells were incubated with anti-CD16/32 Fc block (BD Biosciences, San Jose, CA) at a 1:200 dilution. Cells were stained with DAPI and APC-conjugated anti-CD45 (Biolegend, 103112) for 20 minutes on ice in 1X PBS containing 0.5% bovine serum albumin (BSA). Live CD45^+^ cells were enriched using both BD FACSAria II and BD Fusion cell sorters (UC Irvine Stem Cell Research Center, Flow Cytometry Core), washed once with 0.04% BSA. Individual samples were then labeled with 100 µl of a unique Cell Multiplexing Oligo (CMO), consisting of a Feature Barcode oligonucleotide conjugated to a lipid (10X Genomics Chromium Single Cell 3’ CellPlex Kit), for 5 min at room temperature. Samples were washed twice with 1% BSA in PBS, counted, and samples were reconstituted to a concentration of 1,000 cells/µl and processed by the UC Irvine Genomics High-Throughput Facility (https://ghtf.biochem.uci.edu) for single cell RNA sequencing using the 10X Genomics Chromium Single Cell 3’ platform. A NovaSeq 6000 sequencer was used to perform RNA sequencing (200 cycles), and sequencing reads were processed using the 10X Genomics CellRanger pipeline. For JHMV infected *Cst7-/-* and control groups, cells from 6 mice were multiplexed and pooled for each individual group. For uninfected *Cst7-/-* and control groups, cells from 5 mice per group were used. To ensure adequate CD45 + cell numbers required to run scRNA-sequencing were achieved, all 10 samples from uninfected control and *Cst7-/-* groups were multiplexed and pooled together. Sequencing data from the *Cst7-/-* and control groups were then separated out using the 10X Genomics’ Cell Ranger pipeline.

### scRNAseq analysis

Using Seurat version 4.1.1, experimental groups were filtered, integrated, normalized, and scaled. Genes that were found in less than 10 cells, as well as cells with less than 500 and greater than 3,500 detected genes, were removed. Additionally, cells with more than 30,000 unique molecular identifiers (UMIs) and greater than 5% mitochondrial counts were regressed out. Gene counts per cell were normalized by total expression, multiplied by a scale factor of 10,000, then transformed to log scale. Resulting cells numbers per group were as follows: control = 3,347 cells; *Cst7-/-* = 3,371 cells; control, JHMV = 3,840 cells; *Cst7-/-*, JHMV = 3,350 cells. Downstream analysis was performed in Seurat, following principal component analysis (PCA) and uniform manifold approximation and projection (UMAP) dimensional reduction [43, 44]. Gene expression signatures of CD45^+^ cells from spinal cords of uninfected and JHMV-infected *Cst7-/-* and control mice at day 21 p.i. were scrutinized and cells from the aggregated dataset were clustered into corresponding immune cell populations by a shared nearest neighbor (SNN) modularity optimization-based clustering algorithm via the FindClusters function in the Seurat analysis package in R. The resulting clusters were defined by analysis of expression levels and distribution of population-specific immune cell markers. Once the clusters were established and identified, plots were generated using Seurat and ggpubr R packages [45].

### Statistical analysis

GraphPad Prism was used to perform statistical analyses. Data for each experiment is presented as mean *±* standard error of mean (SEM) or standard deviation (SD). Values from experimental groups were compared using unpaired (two-tailed) t-test, Mann-Whitney *U* test, or Welch’s t-test. Wilcoxon test was used for analyzing gene expression in scRNAseq clusters, and the resulting *p* values were corrected for multiple comparisons by Holm-Sidak method with a *p* value of ≤ 0.05 considered statistically significant.

## Results

### JHMV infection and *Cst7* expression

**Fig. 1:**
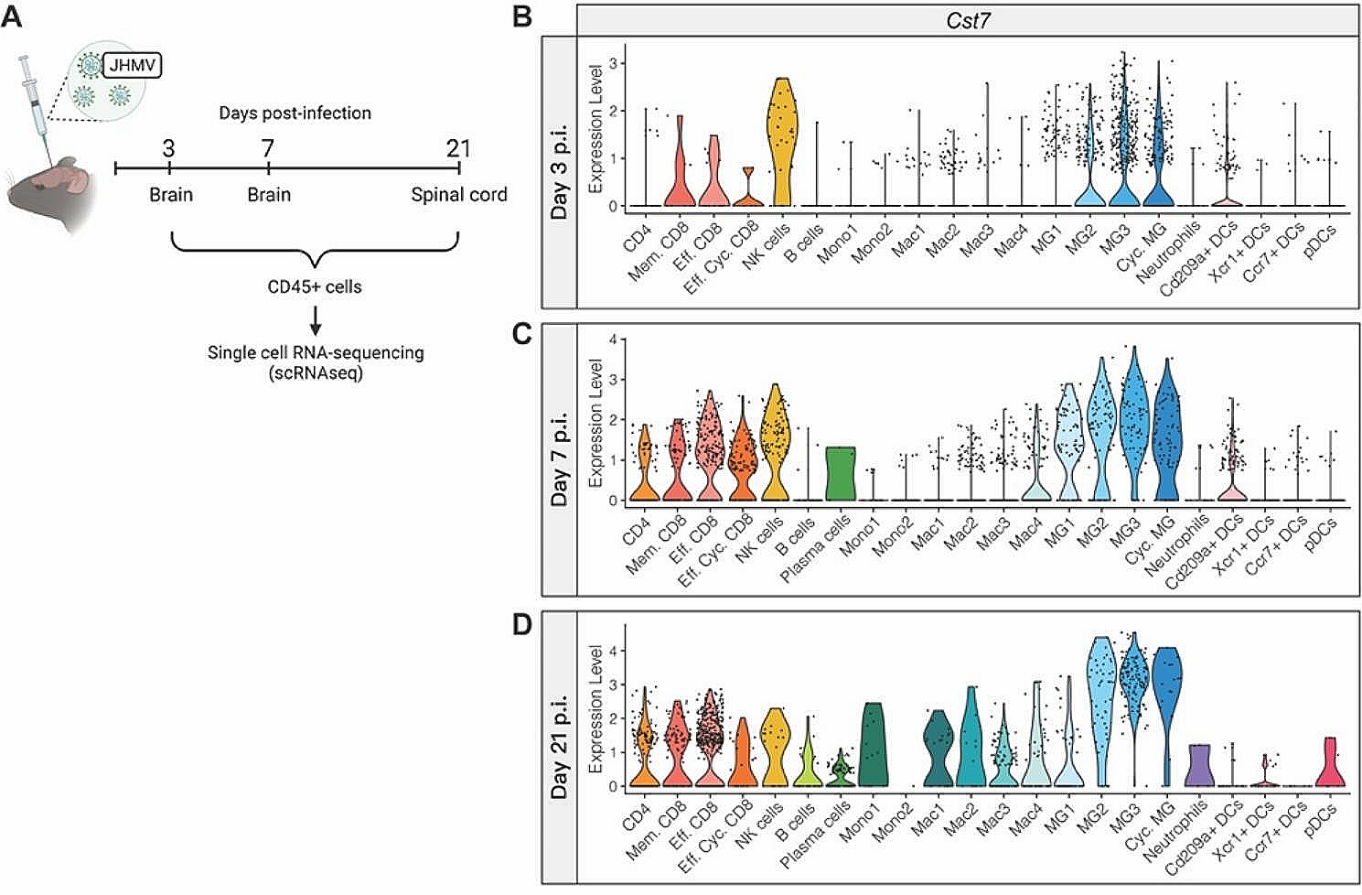
Expression profile of *Cst7* transcripts in CD45 + cells from JHMV-infected mice. (**A**) scRNAseq was conducted on CD45 + cells from brains or spinal cords of JHMV-infected mice at defined times post-infection (p.i.) [29]. Violin plots showing expression levels of cystatin F (*Cst7*) within each cell cluster at (**B**) day 3 p.i. (brains), (**C**) day 7 p.i. (brains), and (**D**) day 21 p.i. (spinal cords). Dots shown in plots ***B-D*** represent individual cells

To determine the mRNA expression profile of cystatin F and its target, cathepsin C, we looked at expression patterns in our previous scRNAseq dataset with adult C57BL/6J mice, infected i.c. with JHMV at days 3, 7, and 21 p.i., from both brains and spinal cords [29] (Fig. 1A). At days 3 and 7 p.i., *Cst7* expression within the brain was almost exclusively restricted to lymphocytes, including CD4+, CD8 + T cells, NK cells, as well as microglia subpopulations (Fig. 1B, C**)**. By day 21 p.i., expression of *Cst7* within the spinal cords of infected mice was much higher than at earlier time points and indicated that inflammatory lymphocytes and microglia, and to a lesser extent macrophages, remained the primary source for *Cst7* transcripts (Fig. 1D). Our finding that microglia were the dominant cellular source of cystatin F transcripts within the brains and spinal cords of JHMV-infected mice is consistent with reported expression patterns of cystatin F within the context of pre-clinical animal models of demyelinating diseases [31–34]. Cystatin F can target cathepsin C and L which regulates various immune cell functions including expression of cytokines. Expression of transcripts specific for cathepsin C (*Ctsc*) and cathepsin L (*Ctsl*) in immune cell populations largely mirrored expression of *Cst7* with microglia, macrophages, and T cells being the prominent cellular sources expressing these transcripts (Supplemental Fig. 1).

### Ablation of *Cst7* does not increase acute JHMV-induced disease

**Fig. 2:**
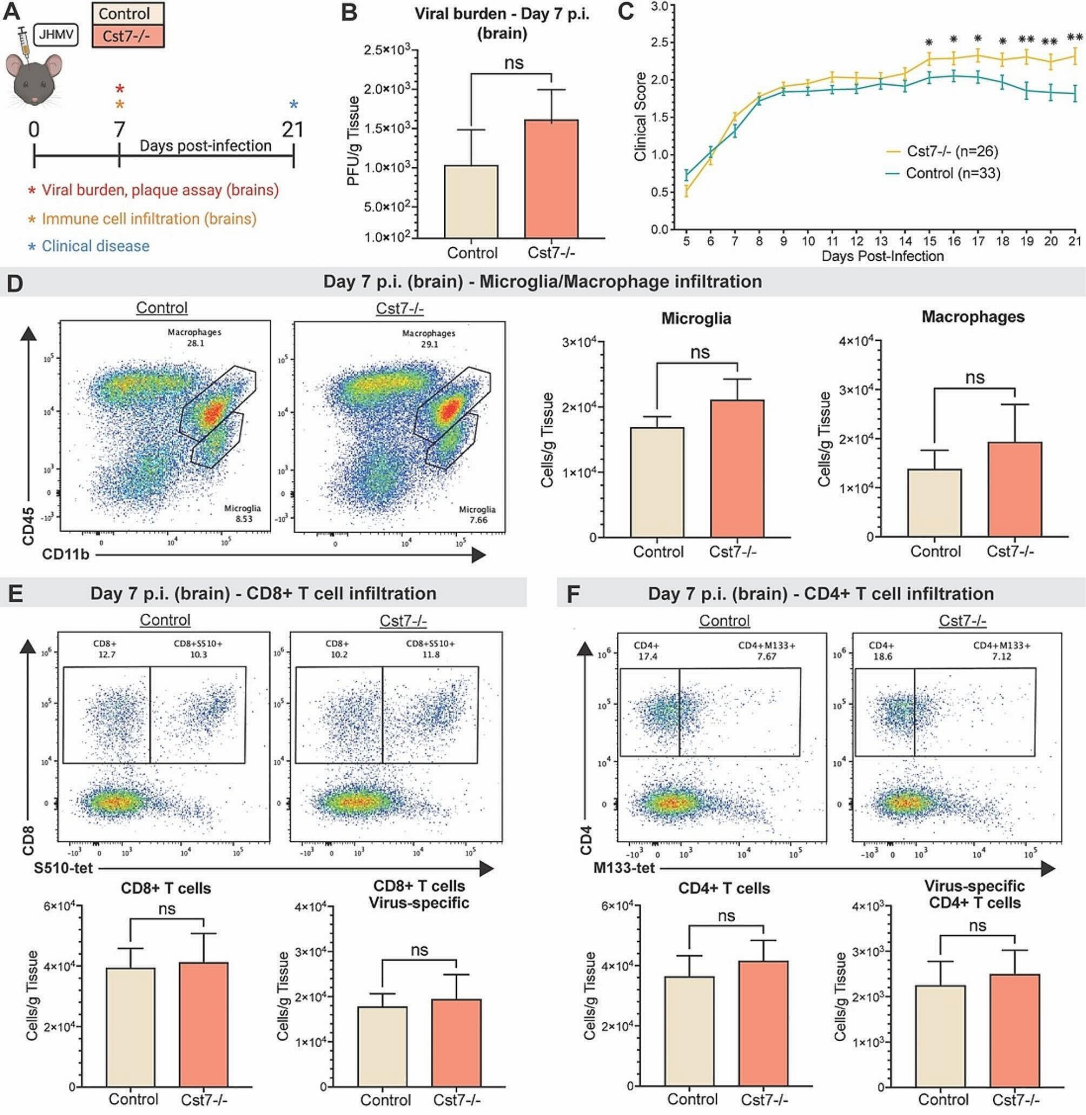
Cystatin F does not play a prominent role during acute disease following JHMV infection. (**A**) *Cst7*^*−/−*^ mice and *Cst7*^*+/+*^ littermate controls were i.c. infected with JHMV. Brains were isolated at day 7 p.i. for flow cytometric analysis and viral titers (*n* = 7–9), and clinical disease was recorded. (**B**) Viral titers on brains at day 7 p.i. showed no significant difference between *Cst7-/-* (*n* = 7) and control mice (*n* = 9); level of sensitivity of plaque assay is ~ 100 PFU/g tissue (**C**) Increased clinical disease from day 15 to 21 p.i. was marked by a more severe hind-limb paralysis in *Cst7-/-* mice (*n* = 26) compared to controls (*n* = 33). (**D**) Representative flow cytometric plots show microglia (CD45^lo^CD11b^+^) and infiltrating macrophage/myeloid cells (CD45^hi^CD11b^+^) in the brains of infected *Cst7-/-* (*n* = 8) or control mice (*n* = 6). Quantification of flow data indicates no difference in microglia or macrophage/myeloid cell numbers. (**E**) Representative staining for CD8 + and S510-518 tetramer revealed no difference in infiltrating CD8 + T cells and virus-specific CD8 + T cells between infected Cst7-/- and controls. (**F**) Staining for CD4 + and M133-147 tetramer showed similar numbers of infiltrating CD4 + T cells (*p* = 0.66) and virus-specific CD4 + T cells between infected groups. Data are presented as average ± SEM; ns - not significant; *p < 0.05, **p < 0.01

*Cst7-/-* and control mice were infected i.c. with JHMV to measure viral burden within the CNS and evaluate clinical disease severity (Fig. 2A). Infected control and *Cst7-/-* mice did not exhibit significant differences in viral titers at day 7 p.i., indicating that control of viral replication was not dramatically altered at this timepoint (Fig. 2B). However, JHMV infection did result in more severe clinical disease in *Cst7-/-* mice compared to control mice beginning at day 15 p.i., which remained more severe out to day 21 p.i. (Fig. 2C). Cystatin F has been suggested to play a role in suppressing target cells from secreting immune cell chemoattractants [34, 46]. To determine if loss of *Cst7* function affected immune cell infiltration into the CNS, we performed flow cytometric analysis on brains of JHMV-infected mice at day 7 p.i. Representative gating for microglia (CD11b^+^CD45^lo^) and macrophages/myeloid cells (CD11b + CD45^hi^) is shown in Fig. 2D. Using this approach, we found no significant difference in numbers of either microglia or macrophages/myeloid cells between control and *Cst7-/-* mice (Fig. 2D). We next looked at CNS inflammatory CD4 + and CD8 + T cells and virus-specific CD4 + and CD8 + T cells via tetramer staining (Fig. 2E, **F**). We did not detect significant differences in infiltration of any of these cell populations between control and *Cst7-/-* mice (Fig. 2E, **F**). These results indicate that cystatin F is not crucial in host defense during acute JHMV infection, as determined by control of viral replication and immune cell infiltration into the CNS.

### Cystatin F and neuroinflammation in mice persistently infected with JHMV

**Fig. 3:**
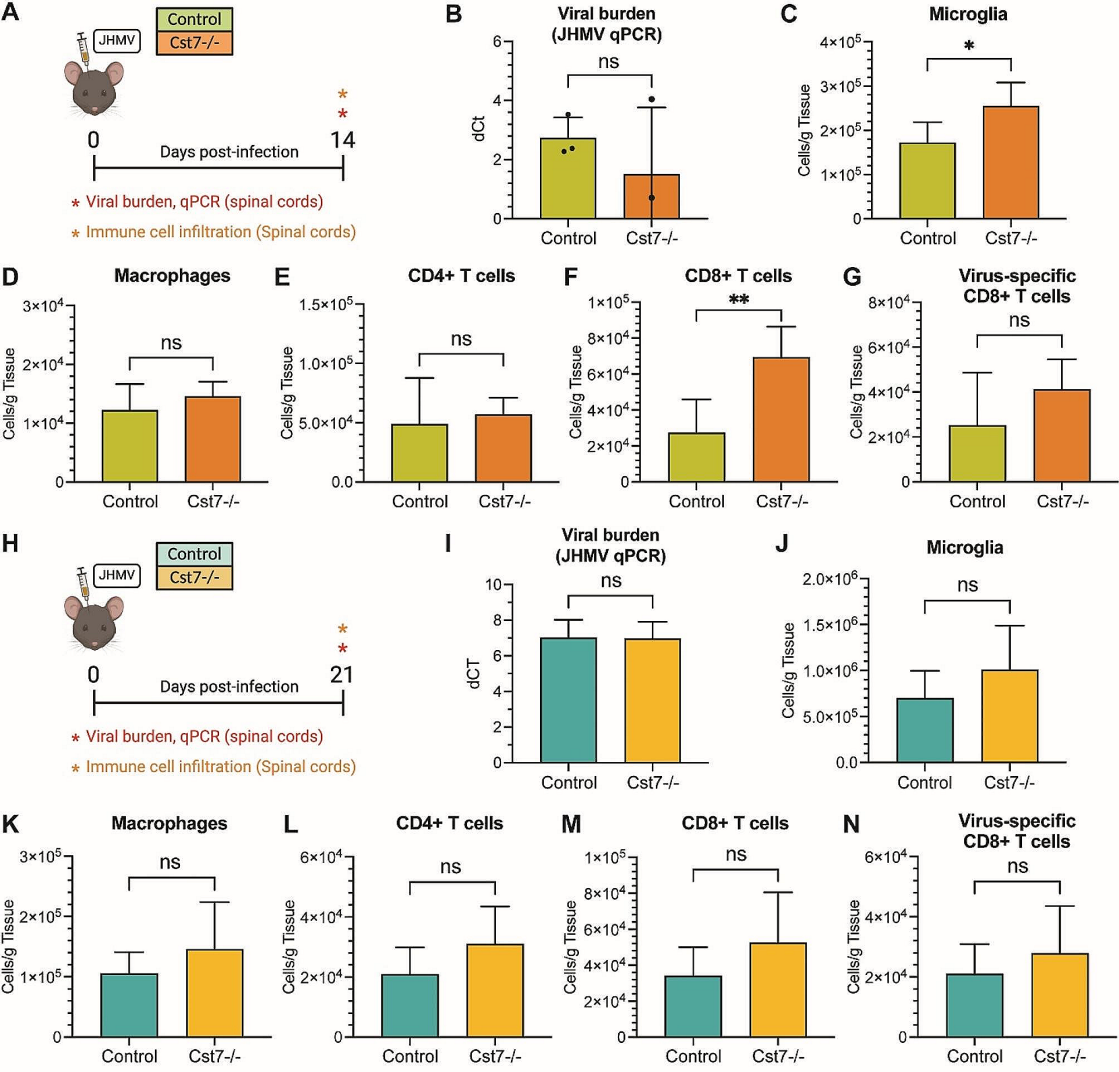
Silencing of Cst7 enhances immune cell infiltration into the spinal cord during early chronic disease following JHMV infection. (**A**) *Cst7-/-* and control mice were i.c. infected with JHMV, and spinal cords were isolated at day 14 p.i. to evaluate immune cell infiltration and viral burden. (**B**) There were no differences in viral RNA between infected *Cst7-/-* mice (*n* = 3) and controls (*n* = 3) at day 14 p.i. Quantification of flow cytometric data from spinal cords comparing (**C**) microglia (CD45^lo^CD11b^+^), (**D**) macrophage/myeloid cells (CD45^hi^CD11b^+^), (**E**) CD4^+^ T cells, (**F**) CD8^+^ T cells, and (**G**) virus-specific CD8^+^ T cells (CD8^+^S510-518 tetramer^+^) between control (*n* = 5) and *Cst7-/-* (*n* = 8) mice demonstrates amplified infiltration of CD8 + T cells (*p* < 0.05) and microglia (*p* < 0.01) in *Cst7-/-* mice at day 14 p.i. (**H**) *Cst7-/-* and control mice were i.c. infected with JHMV, and spinal cords were isolated at day 21 p.i. to evaluate immune cell infiltration and viral burden. (**I**) No differences in viral RNA between infected *Cst7-/-* mice (*n* = 5) and controls (*n* = 5) were found at day 21 p.i. Quantification of flow cytometric data from spinal cords comparing (**J**) microglia, (**K**) macrophage/myeloid cells, (**L**) CD4^+^ T cells, (**M**) CD8^+^ T cells, and (**N**) virus-specific CD8^+^ T cells between control and *Cst7-/-* animals demonstrates no differences in immune cell populations in *Cst7-/-* mice at day 21p.i., (*n* = 5). Data are presented as average ± SD; ns - not significant; *p < 0.05; **p < 0.01

JHMV-infected control mice and *Cst7-/-* mice were sacrificed at days 14 and 21 p.i. to assess effects of silencing cystatin F expression on viral burden and neuroinflammation within the CNS (Fig. 3A, H). qPCR analysis of viral RNA showed no differences in transcript levels between *Cst7-/-* mice and control mice in the spinal cords at day 14 p.i. (Fig. 3B). Moreover, viral RNA levels within the spinal cord were lower in the spinal cords of both Cst7-/- and control mice at day 21 p.i. and no differences in viral RNA levels detected (Fig. 3I). These findings further support the notion that cystatin F is not required for immune-mediated control of JHMV replication within the CNS. Flow cytometric analysis of spinal cords at day 14 indicated increased numbers of microglia (*p* < 0.01, Fig. 3C**)** and total CD8 + T cells (*p* < 0.05, Fig. 3F) in *Cst7-/-* mice compared to control mice yet there were no differences in either macrophages (Fig. 3D), CD4 + T cells (Fig. 3E), or virus-specific CD8 + T cells (Fig. 3G) between *Cst7-/-* mice or control mice. By day 21 p.i., no differences were observed between *Cst7-/-* mice and control mice with regards to microglia, macrophages or inflammatory T cells (Fig. 3J-N).

### Cystatin F influences the severity of demyelination and remyelination in JHMV-infected mice

**Fig. 4:**
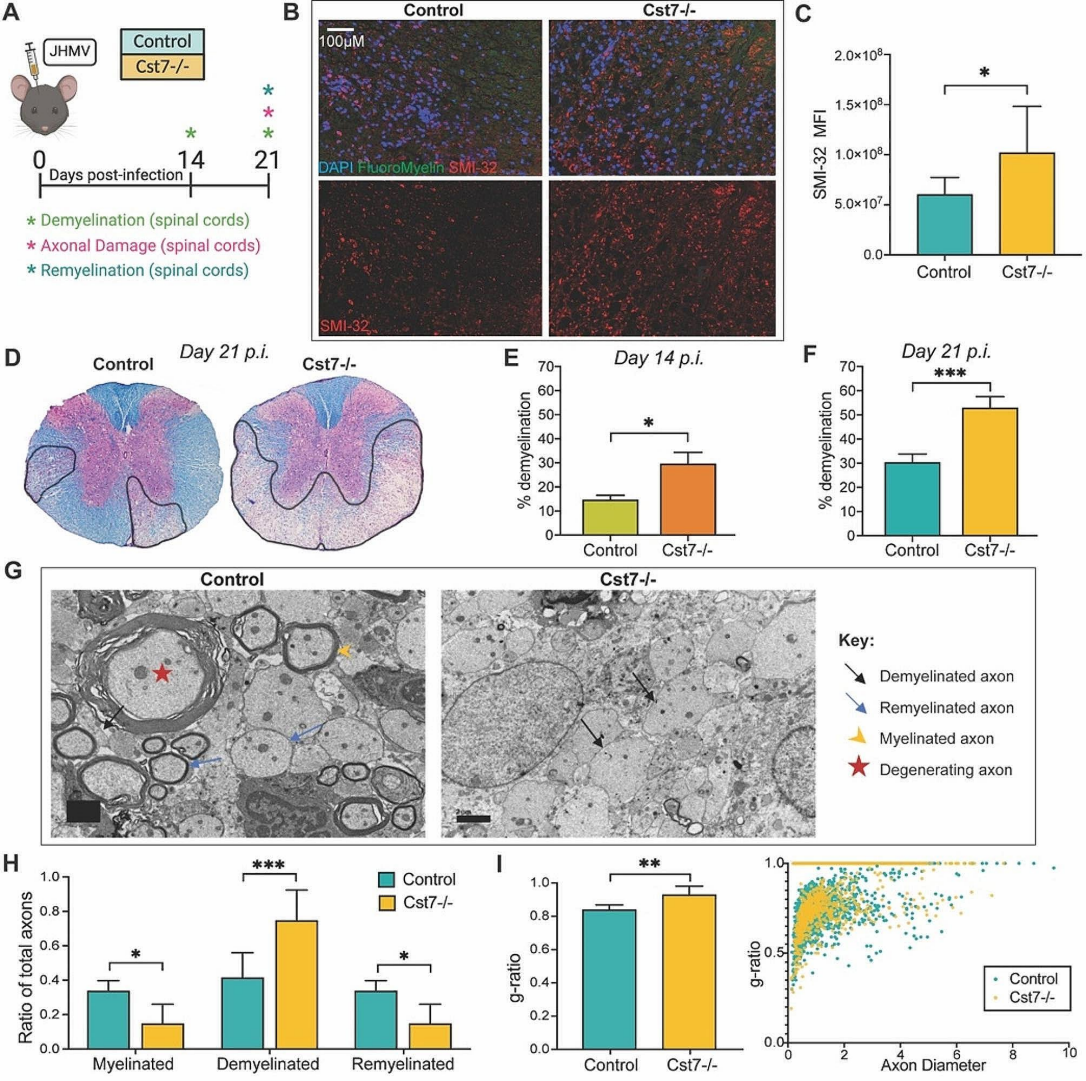
Cystatin F is necessary for remyelination following JHMV-induced demyelination. (**A**) *Cst7*^*−/−*^ mice and littermate controls were i.c. infected with JHMV, and spinal cords were isolated at days 14 and 21 p.i. (**B**) Representative images showing increased SMI32 staining in spinal cords of JHMV-infected *Cst7-/-* mice compared to control mice; upper panels display DAPI (blue), fluoromyelin (green), and SMI32 (red); lower panels show only SMI32 staining at day 21 p.i. (**C**) Quantification of SMI32 staining in *Cst7-/-* mice (*n* = 7) and control mice (*n* = 7). (**D**) Representative spinal cord tissue stained with H&E and LFB (stains myelin fibers blue) from infected *Cst7-/-* and control mice at day 21 p.i., revealing demyelination within the ventral funiculus and lateral white matter columns (outlined by black line). Quantification of spinal cord demyelination at (**E**) day 14 p.i. (*n* = 4, control; *n* = 6, *Cst7-/-*) and (**F**) day 21 p.i. (*n* = 15, control; *n* = 12, *Cst7-/-*) between control and *Cst7-/-* mice. (**G**) Electron micrographs showing ultrathin spinal cord sections from control and *Cst7-/-* mice at day 21 p.i. highlighting myelinated, demyelinated, remyelinated axons and degenerated axons. Histological comparisons in the ventral funiculus and lateral white matter columns between *Cst7-/-* mice (*n* = 6) and controls (*n* = 6) were made from electron micrographs, including (**H**) ratios of myelinated, demyelinated axons, and remyelinated axons calculations, and (**I**) g-ratios (axon diameter/total fiber diameter), Scale bar for ***F*** is 2 μm. For ***H and I***, 133–660 axons were analyzed per mouse. For SMI-32 analysis, data represents mean *±* SD. All other data represent the mean ± SEM; *p < 0.05, **p < 0.01, ***p < 0.001

Spinal cords were removed from uninfected and JHMV-infected *Cst7-/-* and control mice at days 14 and 21 p.i. to evaluate axonal damage and the severity of demyelination (Fig. 4A). Axonal damage in spinal cords was evaluated using SMI32 staining [47, 48] and revealed increased (*p* < 0.05) levels of damage in *Cst7-/-* mice compared to control mice at day 21 p.i. (Figs. 4B, **C**). LFB staining of spinal cords from uninfected *Cst7-/-* mice showed no difference in myelin formation compared to uninfected control mice (**data not shown)**. Compared to control mice, JHMV infection of *Cst7-/-* mice resulted in an increase in spinal cord demyelination at days 14 (*p* < 0.05) and 21 (*p* < 0.001) (Fig. 4D-F). Electron microscopic (EM) analysis of spinal cord sections was performed to evaluate if remyelination was impacted by the absence of cystatin F. High magnification (1200X) images of the ventral and lateral funiculus of spinal cords of JHMV-infected *Cst7-/-* mice, compared to controls, at day 21 p.i. were used to assess differences in remyelination. Measurement of *g*-ratio, the ratio of the inner axonal diameter to the total outer fiber diameter, is commonly employed as a structural index of remyelination, with lower ratios indicating more extensive myelination [36, 37, 49]. Representative EM images revealed an overall increase in demyelinated axons, with accompanying decreased remyelination in *Cst7-/-* demyelinated axons, with accompanying decreased remyelination in *Cst7-/-* mice compared to control mice (Fig. 4G). *Cst7-/-* mice exhibited a significant (*p* < 0.05) decrease in the ratio of myelinated and remyelinated axons and an increase in the ratio (*p* < 0.001) of demyelinated axons compared to infected control mice (Fig. 4H). These data correlated with the increased (*p* < 0.01) *g*-ratios in spinal cord axons from infected *Cst7-/-* mice compared to control mice, further indicating that remyelination is impaired in the absence of cystatin F (Fig. 4I).

### scRNAseq reveals immune cell diversity between *Cst7-/-* and control mice following JHMV infection

**Fig. 5:**
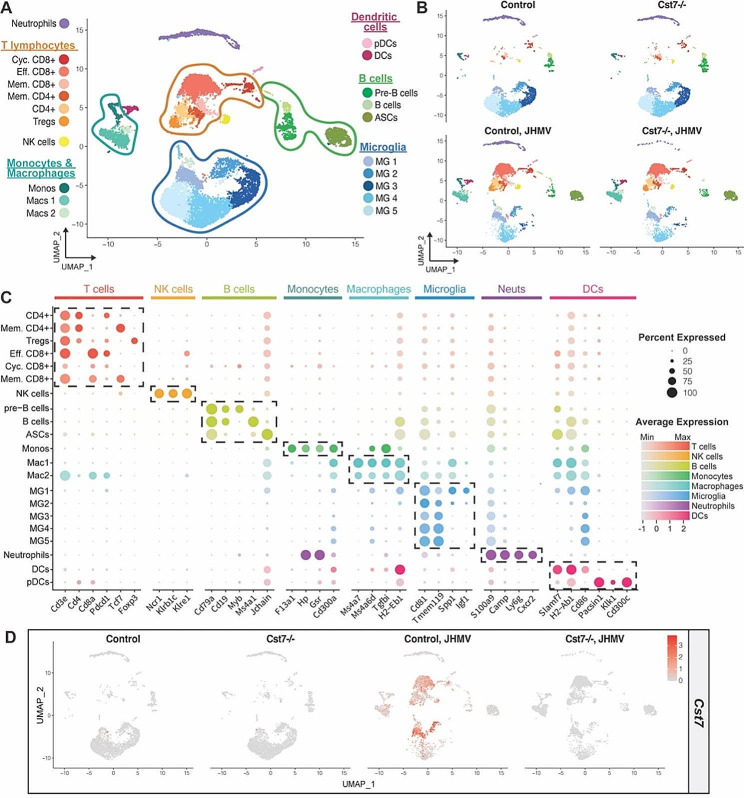
ScRNAseq reveals the immunological landscape within spinal cords of JHMV-infected *Cst7-/-* mice during chronic disease. *Cst7*^*−/−*^ mice (*n* = 6) and controls (*n* = 6) were i.c. infected with JHMV, spinal cords from JHMV infected and uninfected mice were isolated day 21 p.i., and CD45 + cells were sorted for scRNAseq. (**A**) UMAP plot of scRNAseq data revealing 21 distinct cell clusters (aggregate data from uninfected and JHMV-infected *Cst7-/-* mice and controls at day 21 p.i.). (**B**) UMAP plots showing the immune landscape in uninfected and JHMV-infected *Cst7-/-* and control mice at day21 p.i. (**C**) Dot plot presenting expression of cell type specific markers within the 21 cell clusters. Dot size is representative of the percentage of cells from the cluster that express the gene, while the degree of color intensity exhibits the average expression level of the gene. The dashed boxes highlight commonly and uniquely expressed genes of clusters within overarching cell types. (**D**) Expression pattern and levels of *Cst7* transcripts across each sample

To better understand the molecular mechanisms by which cystatin F impacts neuroinflammation and demyelination, we performed scRNAseq on spinal cords of JHMV-infected *Cst7-/-* and wildtype littermate control mice at day 21 p.i. CD45 + cells were flow sorted and enriched, and subsequently Cell Multiplexing Oligo (CMO) labeled and pooled before undergoing scRNAseq. The total number of cells from uninfected control (3,347 cells) and *Cst7-/-* (3,371 cells) and JHMV-infected control (3,840 cells) and *Cst7-/-* (3,350 cells) groups were used for unbiased clustering analysis that produced 21 unique cell clusters in combined uninfected and infected mice (Fig. 5A, B). To corroborate the algorithm-assisted identification of clusters, we assessed expression of known cellular markers in our dataset and found that expression of these markers corresponded with respective cell cluster identities (Fig. 5C). Uninfected control mice showed limited numbers of microglia subpopulations expressing *Cst7* transcripts, while *Cst7* expression was undetectable in *Cst7-/-* mice, confirming the *Cst7*^*em1Aduci*^ allele as being null (Fig. 5D). *Cst7* transcripts were detected in microglia/macrophage and T cells populations in the spinal cords of JHMV-infected mice at day 21 p.i. but were again not detected in *Cst7-/-* mice, as expected (Fig. 5D). These findings indicate that low levels of *Cst7* transcripts are present within the CNS of healthy uninfected mice and are increased under neuroinflammatory conditions following JHMV infection.

### Loss of *Cst7* function produces altered expression of genes related to phagocytosis and remyelination following JHMV infection

**Fig. 6:**
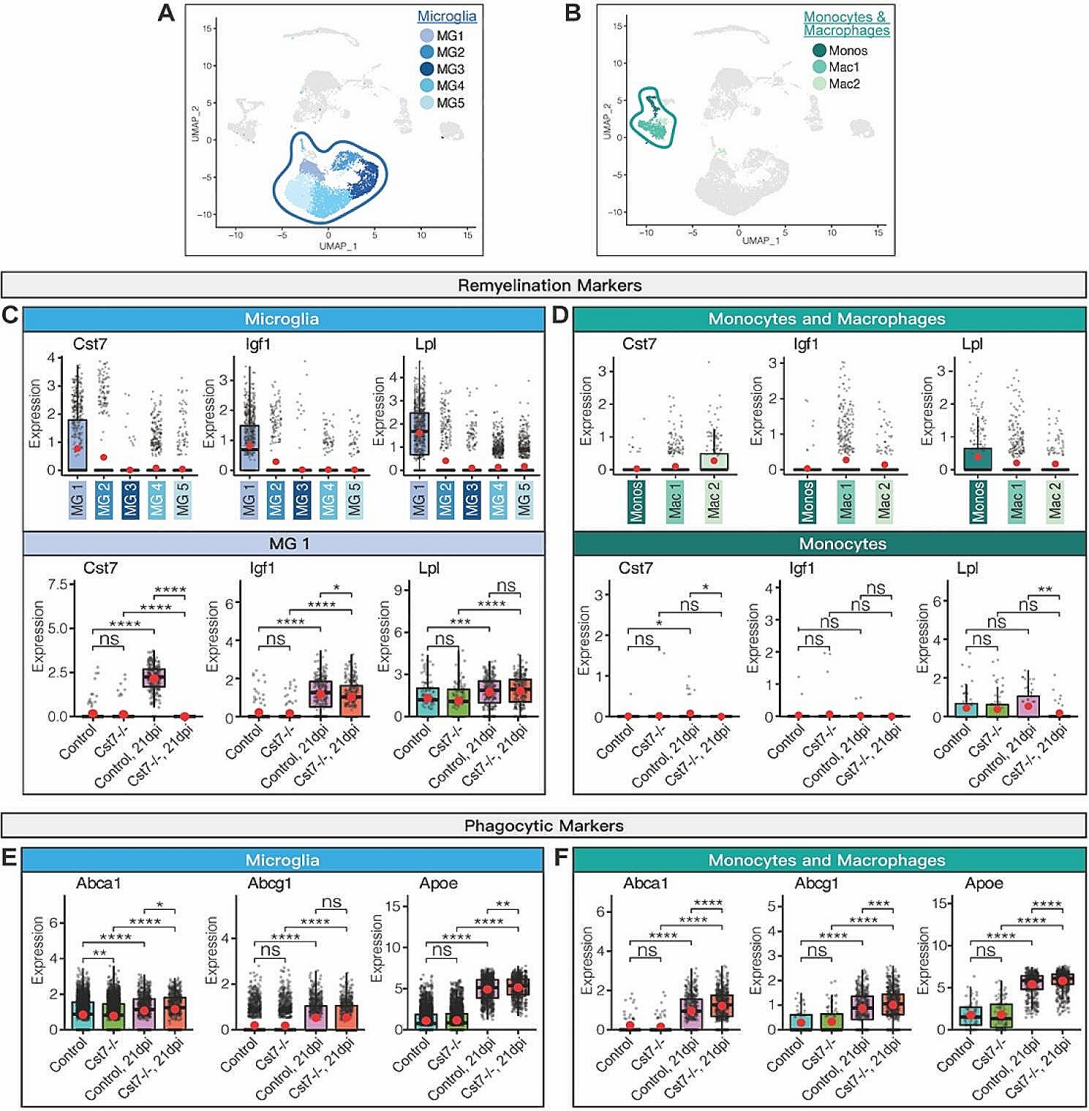
Cst7 ablation produces altered expression of genes related to remyelination and phagocytosis following JHMV infection. UMAP plots highlighting (**A**) microglia and (**B**) monocyte/macrophage clusters. Expression of transcripts encoding remyelination markers cystatin F (*Cst7*), insulin-like growth factor-1 (*Igf1*), and lipoprotein lipase (*Lpl*) in (**C**) microglia and (**D**) monocytes/macrophages. For ***C*** and ***D***, top rows show comparisons between clusters, while bottoms rows compare between uninfected and JHMV-infected *Cst7-/-*, and control mice, in specific clusters. Expression of phagocytic markers was examined in uninfected and JHMV-infected *Cst7-/-* and control mice, revealing an overall increase in expression of *Abca1, Abcg1*, and *Apoe* (apolipoprotein-E) in infected *Cst7-/-* compared to controls in both (**E**) microglia and (**F**) monocyte/macrophage clusters. In ***C-F***, normalized expression values were used, and random noise was added. Box plots show interquartile range, median value (bold horizontal bar), and average expression per sample (red dot). Wilcoxon test was used; ns (not significant) *p* > 0.05, *p < 0.05, ** *p* < 0.01, *** *p* < 0.001, ****p < 0.0001

We have previously reported increased transcription of genes associated with remyelination in the spinal cords of JHMV-infected mice, including *Cst7*, *Igf1* (insulin growth factor 1), and *Lpl* (lipoprotein lipase). There was one microglia subpopulation (MG1) within the spinal cords of JHMV-infected control mice that expressed all three transcripts (Fig. 6A, C). Further examination indicated decreased expression of *Igf1* transcripts in MG1 cells from *Cst7-/-* mice compared to control mice, while there were no differences in expression of *Igf1* and *Lpl* in MG1 between infected controls and *Cst7-/-* mice (Fig. 6A, C). Expression of these transcripts within inflammatory monocytes and macrophages revealed a broader distribution of expression in subpopulations of these cells; though, neither population of cell was a prominent cellular source of these transcripts as compared to microglia (Fig. 6B, D). Macrophage subpopulations, Mac1 and Mac2, trended to be a more prominent source for *Cst7* and *Igf1* transcripts, respectively, whereas inflammatory monocytes expressed higher levels of *Lpl* (Fig. 6D). Expression of *Lpl* transcripts was reduced (*p* < 0.01) in inflammatory monocytes from infected *Cst7-/-* mice compared to controls (Fig. 6D). These findings indicate that silencing of *Cst7* did not dramatically affect expression of transcripts associated with remyelination in microglia or monocytes/macrophages in JHMV-infected mice, suggesting that alternative mechanisms were associated with increased white matter damage and reduced repair. Expression of transcripts encoding anti-inflammatory mediators, including IL-10 (*Il10*), TGFβ (*Tgfb1*), and Annexin A1 (*Anxa1*), were also evaluated in inflammatory myeloid and T cells. No significant changes in these transcripts were detected in spinal cord microglia (Supplemental Fig. 2A) and monocytes/ macrophages (Supplemental Fig. 2B). For inflammatory CD4 + T cells, no difference in *Anxa1* and *Tgfb1* transcripts were detected yet *Il10* transcripts were increased (*p* < 0.05) in CD4 + T cells in Cst7-/- mice compared to controls (Supplemental Fig. 2C) while no differences in any of these transcripts were detected in inflammatory CD8 + T cells (Supplemental Fig. 2D).

Impaired phagocytosis of myelin debris by myeloid cells has been associated with impaired remyelination in JHMV-infected mice [28, 36, 50]. Therefore, one potential pathway associated with inefficient remyelination in JHMV-infected mice includes reduced phagocytosis of myelin debris by microglia. Within the spinal cords of JHMV-infected *Cst7-/-* mice, there was increased expression of transcripts associated with phagocytosis within spinal cord microglia, including *Abca1* (ATP binding cassette subfamily A member 1, *p* < 0.05) and *Apoe* (apolipoprotein E, *p* < 0.05), compared to infected controls at day 21 p.i. (Fig. 6E), while there were no differences in the number of *Abcg1* transcripts (ATP binding cassette subfamily G member 1) between infected *Cst7-/-* and control mice (Fig. 6E). Within spinal cord monocytes/macrophages, there were increased (*p* < 0.001) of *Abca1*, *Abcg1*, and *Apoe* transcripts in infected *Cst7-/-* mice compared to infected control mice (Fig. 6F). These findings indicate that deletion of *Cst7* increases expression of genes associated with phagocytosis within myeloid cells and that deficient phagocytosis of myelin debris in JHMV-infected *Cst7-/-* mice may not be responsible for deficient remyelination, but instead could be representative of a response to the elevated levels of demyelination found in *Cst7-/-* mice.

### Increased transcripts of monocyte/macrophage and T cell chemoattractant chemokines in spinal cords of JHMV-infected *Cst7-/-* mice

**Fig. 7:**
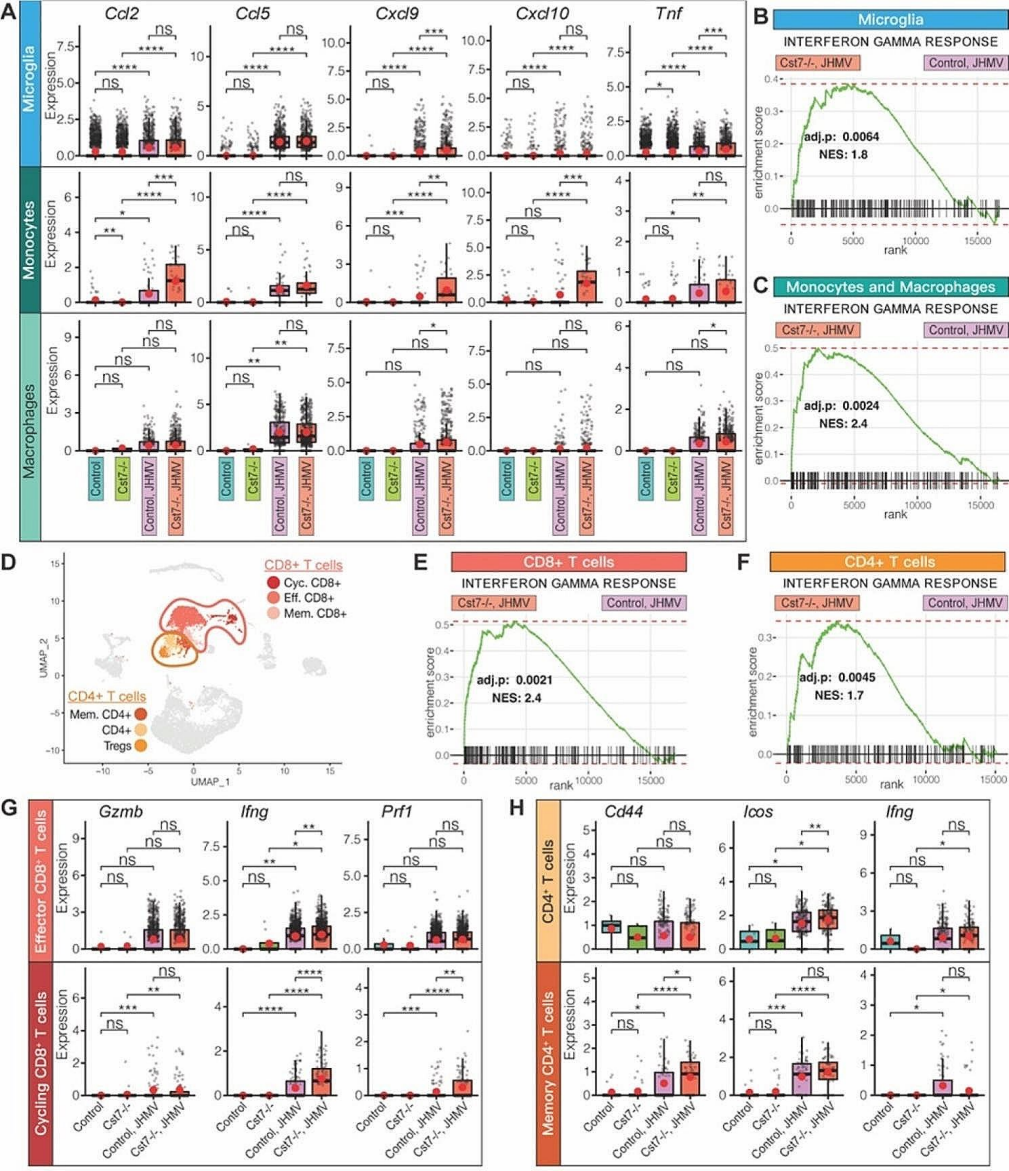
*Cst7* ablation augments T cell activation and chemokine expression. (**A**) Levels of transcripts encoding macrophage chemoattractants, *Ccl2* and *Ccl5*, T cell chemoattractants, *Cxcl9* and *Cxcl10*, and tumor necrosis factor-alpha (TNF-α, *Tnf*) in monocyte, macrophage, microglia populations. Gene set enrichment analysis (GSEA) for IFN-γ responses in combined microglia (**B**) and monocyte/macrophage (**C**) clusters revealed elevated expression of genes related to IFN-γ responses in JHMV-infected *Cst7-/-* mice compared to controls. (**D**) UMAP plot highlighting CD8^+^ T cell clusters (Eff. CD8^+^, Cyc. CD8^+^, Mem. CD8^+^) and CD4^+^ T cell clusters (CD4^+^, Mem. CD4^+^). IFN-γ responses were also amplified in combined CD8^+^ T cells (**E**) and CD4^+^ T cells (**F**) in infected *Cst7-/-* mice compared to controls. (**G**) Eff. and Cyc. CD8^+^ T cells from infected *Cst7-/-* mice have increased expression of CD8^+^ T cell activation markers, granzyme b (*Gzmb*), perforin (*Prf1*), and IFN-γ (*Ifng*), compared with controls. Furthermore, expression levels of CD4^+^ T cell activation markers, *Cd44* (CD44), *Icos* (inducible T cell co-stimulator), and *Ifng* (IFN-γ), were augmented in CD4^+^ T cells and Mem. CD4^+^ T cells from infected *Cst7-/-* mice compared to controls (**H**). In ***A, G-H***, normalized expression values were used, and random noise was added. Box plots shows interquartile range, median value (bold horizontal bar), and average expression per sample (red dot). Wilcoxon test was used; ns - not significant; *p < 0.05, ** *p* < 0.01, *** *p* < 0.001, ****p < 0.0001

We have previously shown that chemokines associated with macrophage and T cell recruitment contribute to increased demyelination in JHMV-infected mice [49, 51, 52]. Cystatin F has been linked with influencing genes associated with inflammation and regulating T cell effector function [34, 53–56]. Therefore, we next examined if increased demyelination in JHMV-infected *Cst7-/-* mice correlated with dysregulated expression of proinflammatory chemokines, leading to increased T cell and myeloid cell accumulation in the spinal cord. We examined expression of chemokine transcripts encoding monocyte/macrophage chemokines, *Ccl2* and *Ccl5*; T cell chemoattractant chemokines, *Cxcl9* and *Cxcl10*; and *Tnf*-alpha in monocytes, macrophages, and microglia within the spinal cords of infected *Cst7-/-* and control mice at day 21 p.i. The level of transcripts encoding *Ccl2* was significantly (*p* < 0.001) increased in *Cst7-/-* mice compared to control mice in inflammatory monocytes but not macrophages or microglia (Fig. 7A). There was no difference in expression of *Ccl5* in monocytes, macrophages, or microglia between controls and *Cst7-/-* mice (Fig. 7A). Expression of *Cxcl9* was significantly increased in monocytes (*p* < 0.01), macrophages (*p* < 0.05) and microglia (*p* < 0.001) in *Cst7-/-* mice compared to controls (Fig. 7A), while expression of *Cxcl10* was only significantly (*p* < 0.001) increased in monocytes of *Cst7-/-* mice compared to controls (Fig. 7A). *Tnf* transcripts were only elevated in macrophages (*p* < 0.05) and microglia (*p* < 0.001) in *Cst7-/-* mice compared to control mice (Fig. 7A**)**. We also determined that IFN-g response signatures in microglia (*p* < 0.006) (Fig. 7B) and inflammatory monocytes/macrophages (*p* < 0.002) (Fig. 7C) from infected *Cst7-/-* mice were increased in CD8 + T cells (*p* < 0.002) and CD4 + T cells (*p* < 0.005) from *Cst7-/-* compared to control mice (Fig. 7D-F). Expression of IFN-g transcripts was elevated in both effector (*p* < 0.05) and cycling (*p* < 0.001) CD8 + T cells (Fig. 7G) but neither CD4 + T cells or memory CD4 + T cells (Fig. 7H) from infected *Cst7-/-* mice compared to controls. Examination of other markers of CD8 + T cell activation indicated no differences between *Cst7-/-* and control mice in transcripts encoding granzyme B (*Gzmb*) in either effector or proliferating CD8 + T cells (Fig. 7). Expression of perforin transcripts (*Prf1*) was increased (*p* < 0.01) in proliferating CD8 + T cells from *Cst7-/-* mice compared to controls (Fig. 7G). For CD4 + T cells, there was only increased (*p* < 0.05) expression of transcripts encoding activation marker CD44 (*Cd44*) in memory CD4 + T cells from *Cst7-/-* mice, with no differences in transcripts encoding for inducible T cell co-stimulator (ICOS, *Icos*) or IFN-g in either CD4 + T cells or memory CD4 + T cells between *Cst7-/-* and control mice (Fig. 7H). These findings indicate that in the absence of cystatin F, there is enhanced expression of proinflammatory chemokines, associated with increased inflammatory monocytes/macrophages and activated T cells leading to white matter damage.

## Discussion

The ubiquitously expressed cystatin C has received more attention in the realm of neurodegenerative diseases, including Alzheimer’s disease, amyotrophic lateral sclerosis (ALS), and MS, for its inhibitory effect on cathepsin B [57–59]. However, the role of cystatins and their target cathepsins in CNS disease remains a relatively new field of research and not well characterized. Unlike cystatin C, cystatin F is secreted as an inactive disulfide-linked dimer and only becomes active upon proteolytic cleavage of 15 N-terminal residues when endocytosed by target cells, where it is trafficked to endosomal/lysosomal compartments, suggesting that its primary function is to modulate surrounding cells, in addition to acting in an autocrine manner [60–62]. Cystatin F is more commonly known for its use by cancer cells to immunosuppress granule-mediated cytotoxicity of effector CD8 + T cells and NK cells [55, 56, 63, 64].

Cystatin F has recently been found to play a role in modulating disease severity in pre-clinical mouse models of demyelination, including experimental autoimmune encephalomyelitis (EAE), as well as toxin and genetic models of disease [31–34]. In addition, cystatin F has been found at the leading edges of plaques in post-mortem tissue from MS patients, further supporting a potential role in both demyelination and remyelination [31–34]. Expression of *Cst7* transcripts is dramatically reduced in mice in which microglia have been ablated, and this was associated with increased demyelination and reduced remyelination in response to JHMV infection, arguing for a protective role for cystatin F in regulating chronic viral-induced neurologic disease [30]. The current study was undertaken to better understand the functional role of cystatin F in regulating host immune responses following JHMV infection of the CNS, as well as further evaluate its role in influencing neuroinflammation, demyelination, and remyelination using mice lacking *Cst7* function.

Previous studies have shown that microglia are important in aiding in host defense as well as in limiting demyelination and enhancing remyelination in response to JHMV infection [36]. This likely involves a multitude of specialized microglial functions, including by enhancing antigen presentation to CNS infiltrating T cells, phagocytosis of myelin debris, which inhibits OPC differentiation and remyelination [65–69], recruiting and promoting differentiation of OPCs [70, 71], and altering the microenvironment to encourage remyelination and survival of OPCs via secretion of growth factors and signaling mediators [72–76]. Microglia depletion prior to JHMV infection leads to an increase in mortality associated with impaired control of viral replication, decreased expression of MHC class II and muted CD4 + T cell responses highlighting an important role for microglia in host defense following JHMV infection of the CNS [27, 30]. Viral replication within the brains of JHMV-infected *Cst7-/-* was not altered compared to infected control mice and there were no differences in either clinical disease severity or mortality during acute disease arguing that cystatin F does not play a critical role in generation of an effective anti-viral T cell response. Targeted depletion of microglia also leads to increased demyelination and limited remyelination, associated with reduced level of transcripts for *Igf1*, *Lpl*, and *Cst7* [77–79]. Impaired remyelination in JHMV-infected *Cst7-/-* mice was not associated with a dramatic alteration in transcripts encoding either *Igf1* or *Lpl*, indicating that cystatin F may not contribute to remyelination by modulation of remyelination-associated gene expression. More recently, remyelination in JHMV-infected mice has been shown to be related to efficient phagocytosis of myelin debris, and microglia appear to be critical in this process [28, 36, 50]. In the absence of *Cst7* expression in JHMV-infected mice, there was an increase in expression of transcripts associated with myeloid cell phagocytosis/lipid metabolism, including *Apoe*, *Trem2*, and *Cd36*, in the *Cst7-/-* mice compared with controls, which argues against impaired myelin phagocytosis by either microglia and/or inflammatory monocytes/macrophages.

Cystatin F is known to act through inhibiting its target protease, cathepsin C, which cleaves pro-enzymes, including granzymes in CD8 + T and NK cells, which regulates cytotoxicity of these cells [46, 55, 56, 63, 64]. Furthermore, cathepsin C has also been linked with regulating microglia activation and expression of proinflammatory genes [46, 58, 80, 81]. Overexpression of cathepsin C aggravates neuroinflammation in a model of traumatic brain injury (TBI) via increased expression of macrophage chemoattractant chemokine, *Ccl2*, and neutrophil chemoattractant *Cxcl2* [53], and genetic silencing of cystatin F resulted in increased demyelination, associated with increased *Cxcl2* gene expression in the cuprizone model of demyelination [34]. Consistent with these findings are our results indicating increased expression of transcripts encoding proinflammatory chemokines by inflammatory monocytes/macrophages and microglia. Our findings support and extend these earlier studies and demonstrate that, in the absence of cystatin F, there is increased expression of transcripts encoding the monocyte/macrophage attractant chemokine, *Ccl2*, as well as T cell chemoattractant chemokines, *Cxcl9* and *Cxcl10*, in inflammatory monocytes/macrophages and microglia in infected *Cst7-/-* mice compared to controls. Whether this is due to specific roles for cystatin F in influencing expression of these chemokines in myeloid cells is not known and warrants further investigation. We have previously demonstrated an important role for both CXCL9 and CXCL10 in attracting T cells into the CNS of JHMV-infected mice which amplify spinal cord demyelination [8, 49, 82–84]. In addition, CCL2 has an important role in attracting monocyte/macrophages into the CNS that increases demyelination severity [51, 52]. The increase in severity of spinal cord demyelination in JHMV-infected *Cst7-/-* mice was associated with increased numbers of microglia and CD8 + T cells at day 14 p.i. when compared to control mice. While demyelination worsened by day 21 p.i. in infected *Cst7-/-* mice, there were no significant differences in immune cell infiltration within the spinal cords between *Cst7-/-* mice and control mice. However, we did identify increased expression of both IFN-g and perforin transcripts in CD8 + T cells present in the spinal cords of infected *Cst7-/-* mice. With regards to inflammatory T cells, *Ifng* transcripts were increased in both effector and cycling CD8 + T cells in *Cst7-/-* mice yet were not detected in CD4 + T cells or memory CD4 + T cells. Nonetheless, increased expression of CD8 + T cell IFN-g was associated with elevated IFN-g responses by myeloid cells as well as CD8 + and CD4 + T cells, indicating that, in the absence of cystatin F, there is a heightened state of immune cell activation, and this is associated with increased myelin damage. In further support of this are studies from our laboratory and others demonstrating both IFN-g and CXCL10 increase apoptosis of oligodendrocyte progenitor cells, and this would further explain the increased in demyelination and paucity in spinal cord remyelination in JHMV-infected *Cst7-/-* mice [85, 86]. Increased expression of perforin transcripts in proliferating CD8 + T cells from *Cst7-/-* mice suggests enhanced cytolytic activity by these cells. Cystatin F is an important regulator of cytolytic activity in both NK cells and T cells [54, 55], and increased cystatin F levels correlate with reduced CTL cytotoxicity [56]. Increased CTL activity/perforin secretion may also be contributing to the extensive spinal cord demyelination observed in the *Cst7-/-* mice [87, 88]. It is also important to mention that we did not detect changes in transcripts encoding anti-inflammatory cytokines IL-10, TGFb, and Annexin 1. Nonetheless, it is inevitable that germline ablation of *Cst7* affects gene expression in other cells types specifically resident cells of the CNS e.g. astrocytes, oligodendroglia and neurons that may have impact neuroinflammation as well as demyelination. We are currently examining the impact of gene expression in these cells following JHMV infection of *Cst7-/-* mice. Collectively, these findings indicate that influencing cystatin F expression within the CNS may offer the opportunity to regulate inflammation and/or pathogenic T cell activity, which may impact the severity of neuroinflammation and demyelination.

### Electronic supplementary material

Below is the link to the electronic supplementary material.

Supplementary Material 1

## Data Availability

All data and materials will be made available upon request to the corresponding author. The *Cst7-/-* mice (C57BL/5J-Cst7^*em1Aduci*^/J, Strain #:037727) have been deposited at The Jackson Laboratory. The RNAseq data will be deposited in NCBI’s Gene Expression Omnibus.
