# Supplementary material for: Cystatin F attenuates neuroinflammation and demyelination following murine coronavirus infection of the central nervous system
Source: J Neuroinflammation. 2024 Jun 15;21:157. doi: 10.1186/s12974-024-03153-0 (PMC11179388; doi:10.1186/s12974-024-03153-0)
Supplement: Supplementary file 1 — Supplementary Material 1 [file 12974_2024_3153_MOESM1_ESM.docx]

**Supplemental Figure 1. Expression profile of *Cstc and Cstl* transcripts in CD45+ cells from JHMV-infected mice.** scRNAseq was conducted on CD45+ cells from brains (days 3 and 7 p.i.) or spinal cords (day 21 p.i.) of JHMV-infected mice (30). Violin plots showing expression levels of Cathepsin C (*Cstc*) and Cathepsin L (*Cstl*) within each cell cluster at (**A**) day 3 p.i., (**B**) day 7 p.i. (brains), and (**C**) day 21 p.i. (spinal cords). Dots shown in plots ***A-C*** represent individual cells.


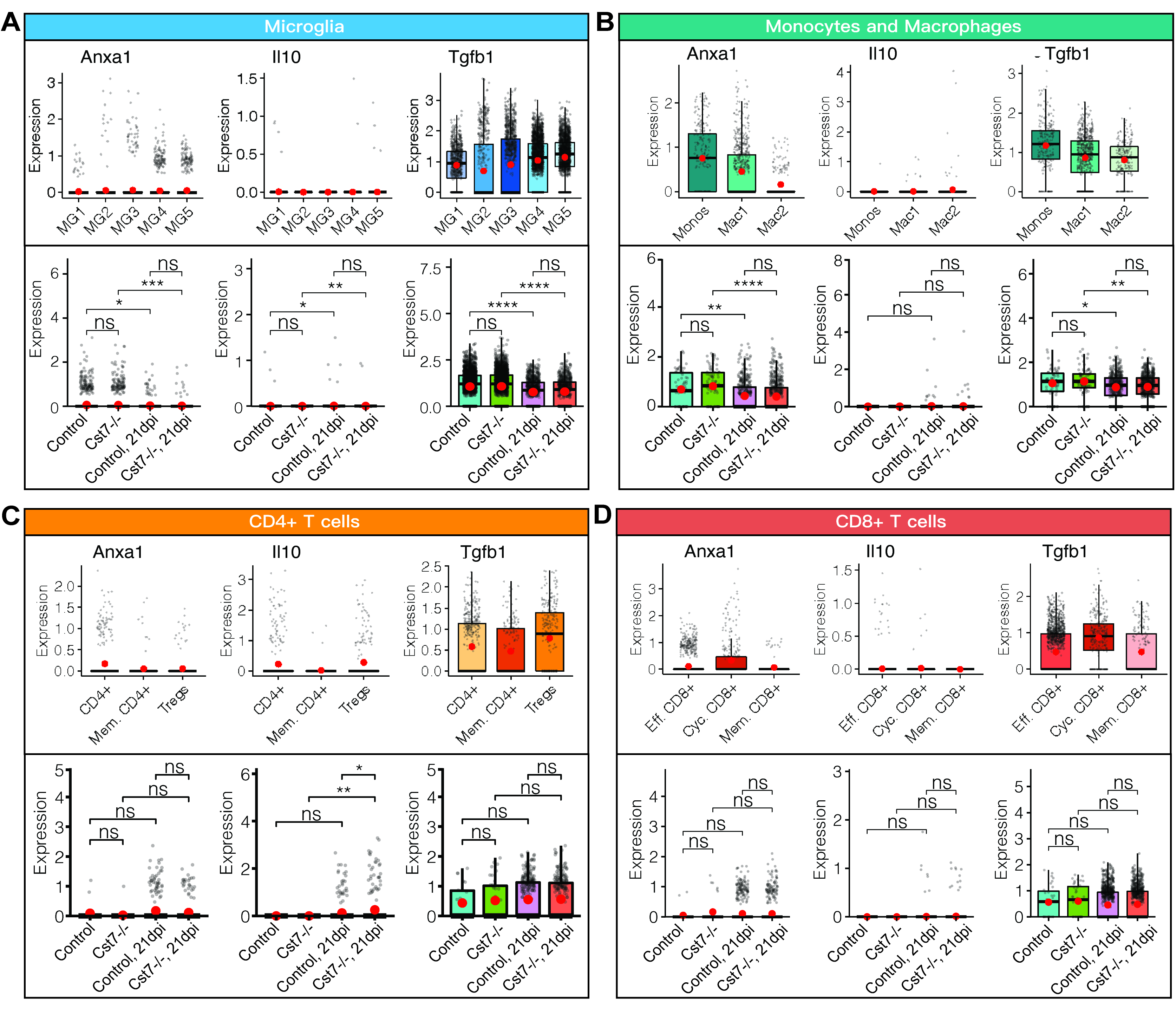


**Supplemental Figure 2. Cst7 ablation does not impact expression of anti-inflammatory genes following JHMV infection**. Expression of transcripts encoding anti-inflammatory markers Annexin 1 (*Anxa1*), IL-10 (*Il10*), and TGF-ß (*Tgfb1*) in combined (**A**) microglia, (**B**) monocyte/macrophage, (**C**) CD4+ T cell, and (**D**) CD8+ T cell populations. For ***A-D****,* top rows show comparisons between clusters, while bottoms rows compare between uninfected and JHMV-infected *Cst7-/-*, and control mice within those combined clusters. In ***A-D***, normalized expression values were used, and random noise was added. Box plots shows interquartile range, median value (bold horizontal bar), and average expression per sample (red dot). Wilcoxon test was used; ns (not significant) p>0.05, *p<0.05, ** p< 0.01, *** p<0.001, ****p< 0.0001.
